# Local health governance in Tajikistan: accountability and power relations at the district level

**DOI:** 10.1186/s12939-020-1143-7

**Published:** 2020-03-02

**Authors:** Eelco Jacobs, Claudia Baez Camargo

**Affiliations:** 1 University of Basel, Petersplatz 1, 4001 Basel, Switzerland; 2 KIT Royal Tropical Institute, Mauritskade 63, Amsterdam, 1092 AD The Netherlands; 3 Basel Institute on Governance, Steinenring 60, 4051 Basel, Switzerland

**Keywords:** Health governance, Accountability, Principal-agent theory, Tajikistan, Political economy, District level

## Abstract

**Background:**

Relationships of power, responsibility and accountability between health systems actors are considered central to health governance. Despite increasing attention to the role of accountability in health governance a gap remains in understanding how local accountability relations function within the health system in Central Asia. This study addresses this gap by exploring local health governance in two districts of Tajikistan using principal-agent theory.

**Methods:**

This comparative case study uses a qualitative research methodology, relying on key informant interviews and focus group discussions with local stakeholders. Data analysis was guided by a framework that conceptualises governance as a series of principal-agent relations between state actors, citizens and health providers. Special attention is paid to voice, answerability and enforceability as crucial components of accountability.

**Results:**

The analysis has provided insight into the challenges to different components making up an effective accountability relationship, such as an unclear mandate, the lack of channels for voice or insufficient resources to carry out a mandate. The findings highlight the weak position of health providers and citizens towards state actors and development agents in the under-resourced health system and authoritarian political context. Contestation over resources among local government actors, and informal tools for answerability and enforceability were found to play an important role in shaping actual accountability relations. These accountability relationships form a complex institutional web in which agents are subject to various accountability demands. Particularly health providers find themselves to be in this role, being held accountable by state actors, citizens and development agencies. The latter were found to have established parallel principal-agent relationships with health providers without much attention to the role of local state actors, or strengthening the short accountability route from citizens to providers.

**Conclusion:**

The study has provided insight into the complexity of local governance relations and constraints to formal accountability processes. This has underlined the importance of informal accountability tools and the political-economic context in shaping principal-agent relations. The study has served to demonstrate the use and limitations of agency theory in health governance analysis, and points to the importance of entrenched positions of power in local health systems.

## Background

Local health governance, and particularly accountability towards citizens has been an object of study and debate since the global health community shifted attention to primary healthcare, and engaged with the good governance and decentralisation paradigms. Already the 1978 Alma-Ata declaration espoused people’s “right and duty to participate individually and collectively in the planning and implementation of their health care” [1]. The Bamako initiative operationalised the declaration by promoting decentralisation of health service financing and management and universal access to health [2]. Around the same time the view that decentralisation of public services, and good governance of these services, including health was essential for their enhanced performance, gained wider traction [3, 4].

Despite increasing attention and the development of multiple analytical frameworks for health governance a need remains to empirically test and validate existing frameworks [5]. Relationships of power, responsibility and accountability between health systems actors and the institutions that shape this are considered central to health governance. Yet, the functioning of local governance is often studied from the perspective of decentralisation or provider accountability towards communities [6–8], while a gap remains in understanding how accountability relations between the multitude of governance actors shape local health systems, particularly in the former Soviet Union and Central Asia. Furthermore, a need for a stronger consideration of the political and contextual factors influencing accountability relations remains [9].

This study aims to address this gap by offering an analysis of principal-agent linkages in the local health system of two districts in Tajikistan. Health governance in Tajikistan, or Central Asia in general, has received little attention, and has mainly been focused at central level reforms, such as the introduction of a Basic Benefit Package of health services (BBP) and the role of international aid in this [10–15]. Fragmentation in health financing regulations and stewardship functions coupled with insufficient donor coordination, leading to a proliferation of vertical and pilot programmes suggests the existence of a patchwork of different health governance arrangements across the country [14]. Closer insight into the way local stakeholders in the health system relate to each other, and how the local context shapes these patterns of power is therefore warranted. To analyse governance relations at the local level in Tajikistan a triadic principal-agent framework is used. This framework allows for an exploration of principal-agent linkages between three sets of actors that are considered to form the heart of health governance: government, providers and citizens. Particular attention is paid to the core components that have been identified to contribute to accountability as a principal-agent relationship.

This paper proceeds as follows. The next section outlines the methods used for this qualitative study, and an elaboration on the choice and assumptions underlying the principal-agent analytical framework of this paper. The following section presents the main health system actors in two distinct districts in Tajikistan and an exploration of their accountability relationships. The implications of the key findings are next discussed in light of the wider literature on accountability and governance, both in terms of the lessons from the Tajik experience and the use of agency theory for health governance analysis. Lastly, the paper offers some concluding thoughts on future entry points for policy, practice and research.

## Methods

### Study design and setting

The study design for this research is a comparison of multiple, embedded case studies [16, 17], which has the potential to explore a phenomenon in the context in which it is embedded, particularly when the contexts is integral component of the study, increasing explanatory power and analytic generalizability compared to a single case study design [18].

Field work was conducted in two districts: one of the Districts of Republic Subordination (*Rayoni Respublikanskogo Podcinenja* or *RRP* in Russian), a region encompassing the former Karotegin or Gharm Oblast (Region) in the East and the Hisor valley in the West, and one district+ in Gorno Badakhshan Autonomous Oblast (Region), commonly abbreviated to GBAO. More detail on the political and socioeconomic context of these study settings is provided in Table 1. The districts are not specifically named to protect informants. Instead the districts will be denoted as the RRP district and the GBAO district.

**Table 1:** Study Settings

The Gorno-Badakhshan Autonmous Region (GBAO) is the largest region of Tajikistan in landmass but the smallest in population. It is dominated by the Pamir mountains, known as ‘the roof of the world’, which have contributed to the region’s historical isolation and the development of a distinct regional identity [19] in linguistic, cultural and religious terms. The majority of the population adheres to the Ismaili faith and speaks one of the Eastern Iranian (Pamiri) languages as opposed to the Western Iranian Tajik, with a Kyrgyz minority in the East of the region. Partly as a result of this isolation, low population density and mountainous geography, the region is less developed socioeconomically compared to the central regions of Tajikistan. Most households rely on subsistence agriculture and remittances from migrated family members for their livelihood. During Soviet times tens of thousands of Badakhshanis, also denoted as Pamiris, were forcibly resettled to the cotton growing plains in the southwestern Qurqontheppa south-region, while positions of authority were mostly filled by non-Badakhsanis, which fostered local resentment towards central authorities [20]. This marginalisation was part of the reason for Pamiris to ally with Gharmi and other regional and ideological groups against the government in Dushanbe and form the United Tajik Opposition during the civil war (1992–1997). Given this historical background, the geographical isolation and cultural distinction from the rest of the country, the region remains more distant from the political centre up to this day. However, key positions continue to be filled through appointment from the centre, ensuring a degree of political control.	
The districts of republican subordination (usually denoted by its Russian acronym RRP) are a collection of districts that are governed directly by the central government. The area stretches horizontally across the middle of the Republic of Tajikistan from the Hisor valley at the border with Uzbekistan around 70 km west of the capital Dushanbe, to the Rasht or Karotegin valley in the east, bordering Kyrgyzstan, hemmed in by mountain ranges in the north and southeast. The region has historically never been a unified territory. Rather, it encompasses mountainous areas in the east that were strongholds of the United Tajik Opposition (Karotegin / Gharm region), and more populous plains in the west that have remained under firm control of the central administration in nearby Dushanbe. The district that forms one of the two study sites in this paper is located in the Hisor valley in the western part of the RRP. Due to its proximity to Dushanbe, intensive cotton production on the irrigated plains, and the presence of the largest aluminium manufacturing plant in Central Asia, Tajikistan’s main industrial asset, the area has been of vital interest to the political and economic elite, receiving most of its capital investments. As an expression of that the Hisor elite was closely allied with those from the Khujand/Leninobod north and the Southern Kulobis that dominated the government by the end of the Soviet period and throughout the civil war in the 1990s [20].	

The districts were purposefully chosen for their difference in proximity and interest to the political centre Dushanbe and the fact that the RRP district featured as one of the pilot districts for the implementation of the BBP reforms, while the GBAO district was excluded from the BBP pilot at the time of research. A key element of the reforms is the introduction of co-payments, with the exemption of vulnerable social and disease groups, which is intended to generate extra revenue [12, 14]. In addition, health service delivery in the two districts was supported by two different external development partners, as will be elaborated in the results section, which was assumed to influence local accountability relations in distinct ways.

### Data collection

This research was part of a wider power and influence study of stakeholders in the health system of Tajikistan at central, district and village levels that took place between May 2010 and December 2011. It is based on primary data collected through key informant interviews (KIIs) and focus group discussions (FGDs) in the two study settings between June and August 2011.

KIIs and FGDs were carried out in person and through telephone/Skype and were conducted in Tajik, Russian or English. A total of 23 KIIs were held in the RRP district, and 17 KIIs in the GBAO district. In addition, 6 FGDs were held with villagers in the GBAO district, and 2 FGDs in the RRP district. An overview of respondents is provided in Table 2. The study further draws on the contextual insights gained from 31 KIIs conducted among governmental, bilateral, multilateral and non-governmental organizations, primarily at central level, which were used for another study [14]. Participants for KIIs were purposefully selected, focusing on those involved in the regulation, supervision, financing and provision of health services in the two districts. A snowball technique was used by asking interviewees to suggest the most relevant stakeholders in the local health system. Health providers were mostly interviewed at the health facilities where they worked. This allowed for visual capture of the conditions in which they operated and observation of interactions with patients and co-workers.

**Table 2:** Overview of KIIs and FGDs

District	Method	Type of interviewees / participants	Number of KIIs / FGDs	Total
RRP district	KIIs	District and municipal level government actors	5	KIIs = 23 FGDs = 2
		Health providers	10	
		Development agency	6	
		Community groups / community leaders	2	
	FGDs	Citizens	2	
GBAO district	KIIs	District and municipal level government actors	5	KIIs = 17 FGDs = 6
		Health providers	4	
		Development agency	5	
		Community groups / community leaders	3	
	FGDs	Citizens	6	

As FGD participants were identified through the main organisations implementing health service delivery programmes in the two districts a certain degree of respondent bias is possible. The cases are therefore not taken as a sample representing community views in the district. Rather, each case is taken to provide insights into the contextual factors, processes and mechanisms underpinning actors’ interactions, which can be used for middle range theory-building and further testing [16, 18, 21].

### Data analysis

A topic guide was used for interviews and FGDs, including questions on the organization, financing and provision of health services, as well as the main challenges and responsibilities in these areas. Detailed notes were taken during each interview by each interviewee and compared afterwards on the same day, which allowed for triangulation and the elaboration of more comprehensive field notes. These notes were used for preliminary analysis during the course of the research. This revealed emerging themes and aided the refinement of interview questions in the process to further explore these themes.

Data analysis, which took place shortly after conclusion of the field work, was based on a framework approach [22], guided by the model for analysing accountability processes as principal-agent relationships between three main health system actors that has been developed and refined by Brinkerhoff and Bossert [23, 24] as an application of agency theory to health systems research. This model allows for an analysis of health system governance actors and their relationships of power, responsibility and accountability, based on an understanding of health governance as a process of principals delegating authority to agents, as shaped by the formal and informal institutions and behavioural patterns. Originating from new institutional economics, agency theory essentially forces a lens on the relationship between incentives and performance [25], and the tensions between the interests of the principal and agents, allowing for the existence of multiple agents in the state-policy implementation [26]. Brinkerhoff and Bossert have distinguished between three main groups of actors in their model for health governance: the state, providers and citizens/clients that act as principals and agents to each other in more or less effective ways. To generate greater understanding of these relationships this article firstly describes the local governance actors in the two districts following the actor categories, and subsequently analyses each dyadic relationship in terms of separate dimensions of accountability, captured in the questions. In terms of citizens’ relationships to the state or providers three core elements, which together form the principal –agent process of citizens’ accountability, will be explored: voice, interpreted as ‘articulating an interest’ [27], answerability and enforceability [23, 28, 29]. These core elements can in themselves be considered an aggregate of five steps in any effective accountability process: delegating a mandate (or voice focused at a task), providing resources, performing on the mandate, providing information and being monitored on the performance, and enforcement of the mandate [30]. These five components will be used to guide the analysis of the principal-agent relationship between state and providers for a more detailed understanding. From the findings a fourth actor category was found to exercise significant power as a principal: development agencies. Their role and relation to the other actor categories is therefore discussed separately.

## Results

### Main actors in the local health system

#### The state

In both districts, formally the most important actors in the health sector are state actors, i.e. the district hospital director, who by definition also manages the most important health provider in the district, and the head of the district financial department. Both in turn are selected and appointed by the district chairman (*Rayon Rais*), who is indirectly assigned to his or her post by the Presidential administration in Dushanbe. Budget allocations to health facilities follow from their joint decisions, based on how much funding has been secured from the Ministry of Finance at the central level. Other important actors that surfaced from interviews were the primary healthcare (PHC) manager, responsible for PHC in the district and the district health team (for which the Russian portmanteau *RaZdrav/GorZdrav* was commonly used), de jure responsible for health planning and supervision in the district. In both districts, as was reported to be not uncommon around the country, the deputy hospital director was appointed as PHC manager at the insistence of the hospital director. This was alleged to foster a sense of loyalty and obligation from PHC manager to hospital director. To facilitate the distribution of funds to health facilities in the mountainous area, municipal mayors in the GBAO district have a responsibility in this, acting as a middle man, but hold no further mandate. Local legislative councils, (*Majli*, or assembly of people’s deputies), exist but although legally endowed to approve budgets and policy, they were not indicated by respondents, including council members (referred to as *deputat* by respondents), to exercise any meaningful authority over the financing or management of local health services.

Although initiated to be fulfilling a central role, the position of the district health team, whose members usually consist of a chairman and an accountant, following appointment by the hospital director, was found to be remarkably weak in both districts. Its authority vis-à-vis the other state actors was found to be low, as its mandate is only vaguely defined, overlapping with the roles of district hospital manager and PHC manager, and it cannot lay claim to its own operational budget. In both districts, district health team representatives claimed to receive no funding to carry out their monitoring, supervisory and planning activities apart from a below-subsistence level salary, which was confirmed by KII at central level. District health team staff indicated that this means that independent travel for monitoring and oversight to rural health facilities is impossible, and even a workspace or office equipment is not covered and up to them to negotiate with the hospital director. Lastly, the district health team does not have any authority on budget planning, which is generally a process of negotiation between PHC manager, hospital director and the rayon’s financial department. This combined picture illustrates how the district health team’s role as a coordinating body balancing the needs of primary and specialized health care is undermined. Reports from health staff and administrators in both districts suggest rivalry between the district health team, PHC and hospital managers over mandates between the different rayon level administration officers. One example that surfaced during interviews with district officials and health staff in the RRP district, was a dispute over the management of ambulances. The PHC manager, hospital director and the district health team all laid claim to this role that was traditionally reserved for the hospital director. In separate interviews the different parties covertly expressed that the other actors were merely interested in this management role as it provided a toll position, i.e. the fee patients pay for the service is creamed off by those who manage the ambulance staff.

#### Providers

The most important health providers in both districts are the district hospital, including its policlinic, and the PHC centres: rural health centres and health houses. The district hospitals also contain a chemist. All facilities are publicly owned and receive most of their highly limited income from public sources, although formal user charges for diagnostic tests and pharmaceuticals exist. There were no formal private providers of care in either district at the time of research, and no informal care providers were mentioned by respondents, which leads to the assumption that private providers play no significant role in health service provision. Health centres are generally staffed with multiple personnel, including a physician, and theoretically better equipped. Health houses would typically be staffed by a nurse only, although in practice the observed difference in terms of equipment and infrastructure between the two types of PHC centres was marginal, particularly in the RRP district. A difference could be observed between the degree to which health providers were equipped and the state of infrastructure between the RRP and health providers in the GBAO district were generally better equipped and in a better infrastructural state. This is probably mainly due to the large investments by the Aga Khan Development Network (AKDN), as described in the sections describing the role of development agencies.

#### Citizens - users

The district citizens are considered the main users of the health services in the two districts, although seasonal workers from other districts might make up a small additional group of users. No activity from formal patient groups was found in the districts, but other associations for collective action were found. Two types of relevant organizations for collective action could be discerned: NGO-induced community-based groups (as explained below) and neighbourhood committees, or mahallas. Although citizen respondents generally expressed closer identification with their mahalla, the community-based initiatives were the only groups that could be found to be playing a meaningful role in exerting citizen influence on the health sector, although this role is limited by the restrictive political environment.

#### Development agencies

A fourth relevant actor category in the local governance of health services emerged from the findings, given their relative weight of their involvement in local health services: external development agencies. In the RRP District a Swiss Development Corporation funded project, project Sino, was operational, and in the GBAO district the Aga Khan Development Network (AKDN) through the Aga Khan Health Services (AKHS) and the Mountain Societies Development Support Programme (MSDSP) provided extensive support. In both cases this support included the rehabilitation of health centres, provision of equipment, awareness-raising activities for citizens around health issues, health staff training, technical support for health facility management and limited monthly health worker salary supplementations (officially termed incentive payments). In addition, the AKDN had instituted a revolving drug fund and community based health funds (micro-insurance) through their own community-based organisations, the Village Organisations (VOs).

### Principal-agent relationships between the main actors

This section elaborates on the principal-agent relations between the main actors that establishes the process of local health governance in the two districts.

#### State –providers

Health providers in the two districts are all state-owned, receive most of their budget from the district’s allocations and its employees are civil servants. Formally, the district’s hospital director, PHC manager and the district health team practice regulatory oversight and supervision over the health providers. The district health team’s lack of means to exercise oversight means the hospital and policlinic directors, i.e. the hospital director and PHC manager, practice oversight over their own management. The lack of separation between funding, purchasing, regulation and provision functions that flows from the diverse functions of hospital directors and PHC managers observed in the two districts and the resultant blurring of the principal-agent relationship between state and providers, appears to benefit the district hospital and policlinics over rural PHC facilities.

In terms of a mandate, a BBP defines the formal scope of services provided in the public facilities, with commensurate co-payment rates. In practice the degree to which this mandate could be performed upon was found to be limited by a severe lack of financial resources, impacting equipment, drugs and skills shortages, albeit to uneven extents.

Although formally 40% of health funding is reserved for PHC in the two districts, the budget is so small that staff from rural health centres and health houses reported neglect by district authorities in terms of funding at the expense of the district’s hospitals and policlinics, which was confirmed by visual observation. Budget allocations follow a line-itemised input logic without flexibility to shift funds between budget lines in case of changing needs or priorities. In practice 80 to 90% of allocated funds for rural PHC centres is used to pay for salaries that are below subsistence level^1^ [31]. In the RRP district the use of co-payment revenue, generated under the BBP regulations, was a source of contention. Because BBP co-payments were limited to diagnostic tests and ambulatory surgery for district residents in PHC, the extra resources these user fees generate were limited. The PHC manager gave the example of an ECG device that could be acquired for the polyclinic after saving up the resources for three months. Rural health facility staff on the other hand consistently complained they saw very little to nothing back of the co-payment revenue that they channelled to the PHC manager on a monthly basis. This could be confirmed when analysing the available information on collected co-payments. As was shown in the PHC financial records, the monthly revenue varied from 0 to 150 TJS per rural health facility, with the district capital’s polyclinic generating over 500 TJS monthly. Due to the budgetary split between PHC and hospital care there was no compensation through monies from co-payments in hospital care, where revenues were much greater. One of the other effects of the low level of funding is that although doctors have been trained to operate various types of medical equipment they reported that they lose those skills because the equipment is not available. This effect was however much more widely reported in the RRP district, due to the greater lack of equipment and general lack of running water or constant electricity supply in the PHC centres, which is necessary to run or maintain the technology.

In terms of provision of information on, monitoring and enforcement of performance, rural health workers from both districts showed extensive paperwork for record-keeping of client visits, but claimed supervisory visits were erratic and, when conducted, largely punitive in character, focused on opportunities for rent-seeking. In the GBAO district rural health workers claimed that the district financial authorities or the mayors only come for inspection with the purpose of checking new equipment given by the AKHS, and demanding a share of revenue, even though formally co-payments are not charged at rural health centre level. When supervisory visits were undertaken by district authorities, rural health centre staff from two health centres reported sanctions they experienced as extortionate (twice a nurse’s monthly wage) for missing equipment, which they claimed to be the result of underfunding from the same district authorities. District authorities claimed lack of funds for transport hampered the conduct of regular supervisory visits, and confirmed applying fines in case of ‘wrongdoing’.

#### Citizens – providers

Health providers in the two districts were found to be strongly positioned towards those seeking care, as public providers hold monopolies in care provision and citizens have little capacity to judge the necessity or quality of clinical practices. In addition, particularly in the GBAO district, health centres are sparsely distributed across a highly mountainous landscape, leaving communities with few other options than their local rural health centre or health house. Although the relationship between providers and citizens is marked by great power asymmetry, which applies to some degree to all health systems, a clear difference could be observed in relation to local primary health care providers versus the district hospital and policlinic. Respondents from FGDs and interviews with village representatives in both districts reported relatively higher appreciation for services delivered by their local PHC centre or health house, compared to the district policlinic and hospital, where informal payments were reported to be rife and experienced to be at extortionate levels. In both cases no formal direct voice mechanisms or institutions between citizens and providers, such as report cards, or village health teams were found or reported on. However, villagers expressed much greater trust in the staff of rural health centres and showed understanding for their underfunded position. Health staff in rural health centres and health houses were more perceived as ‘one of them’, particularly in the GBAO district. As opposed to the informal cash payments in the district policlinics and hospitals, village residents in the GBAO district reported giving products from their plots, such as potatoes, to rural health facility staff as gratitude payments, and in some cases lending a hand in small repairs to the clinic’s building in recognition of the lack of resources these health facilities reportedly receive from the district authorities. Although an element of extortion by health providers in demanding these in-kind payments from patients cannot be ruled out, it appeared that these contributions also formed an informal tool of enforceability and contributed to a greater sense of answerability by rural health centres and houses towards citizens as compared to the district hospital and policlinic.

#### State – citizens

KIIs and FGDs with village residents and representatives suggest the relationship between local district government and the district’s inhabitants, who can be assumed to all be potential users of the health system, is marked by low trust and negligible citizen participation in decision-making. Voice is limited by the lack of an electoral process for key positions in local government and the Soviet legacy of authoritarian leadership. The only participatory governance structure that citizens perceived to play a functional role in their lives is the mahalla, or neighbourhood council. In the absence of effective formal channels of citizen participation, the mahallas and AKDN’s community-based organisations in some cases function as a channel to voice needs and concerns towards and mobilise citizens collectively pool or provide resources for healthcare provision or financing, as documented elsewhere [32].

#### The position of development agencies

Although omitted from the triadic health accountability model, development agencies were found to play an influential and distinct role in local health governance. Through their in-kind contributions to local service delivery and health workers, as well as their community activities they occupy a central position towards health providers and citizens. As a bilaterally funded project and a private, faith-based international non-governmental organisation the most significant development actors in the project cannot be considered local civil society or groups of direct citizen representation (conforming to the category of ‘citizens’). They therefore merit separate consideration.

AKDN’s presence in the GBAO district is in part motivated by the presence of a predominantly Ismaili population in the region, for whom the Aga Khan is their main spiritual leader. Combined with their continuous presence since the years of severe food insecurity and conflict in the mid 1990s, and the wide range of other activities it employs in rural, economic and cultural development this lends the organisation a basic level of cultural-religious legitimacy for people in the region. As a channel and platform for community-based support MSDSP set up VOs across GBAO, and later also elsewhere in the country. Although they can serve to channel people’s concerns to the MSDSP, VOs hold no serious leverage to demand answerability from it.

Project Sino’s intervention in the RRP district started in 2004 with tuberculosis control work and soon after broadened its scope to strengthening primary healthcare and public health sensitisation among communities [33]. In other words, community-based groups were not encouraged to function as platforms for citizen’s voices vis-à-vis either providers or state authorities.

Both project Sino’s and AKDN’s interaction with local (health) authorities takes place through regular meetings (in case of AKDN involving either AKHS or the MSDSP depending on the issue), at which local authorities are consulted on local needs in the health sector and they are informed of the projects progress and new initiatives. Similar to project Sino’s involvement with local health authorities this process is merely consultative or informative in character, as other accountability dimensions were found to be weak or non-existent. No financial resources were reportedly being provided directly to local government actors, they were not found to be active participants in AKDN or Sino activities, and no specific performance agreement or reporting obligation between the two actors was found.

The engagement between AKDN and Project Sino with health providers on the other hand bears strong features of a principal-agent relationship. The resources (monetary, in-kind and through capacity-building) provided by the both agencies are directly linked to the general objective and mandate to strengthen primary healthcare and reward health workers, and their use is monitored in relation to provider performance. However, the large dependence of health facilities on this support, implies the external actors have come to compete with the state authorities as their most important principal. This was perceived more strongly in the case of the AKDN in GBAO, where even the interviewed mayors and district administrators considered the AKDN to have partly replaced the state in its role of providing basic services. The consensus was that although basic salaries of health staff are paid by the state the AKHS provides most of the public health campaigns, infrastructure maintenance, retraining of staff, and supply of medicines and technology. One top-level official of the GBAO district even went so far as to call health staff ‘volunteers’ since they perform their duties for a below subsistence-level wage.

## Discussion

This study has provided an understanding of the nature of principal-agent relationships in the local health sector of two districts in Tajikistan and their underlying power dynamics. Beyond that the findings from the two settings can serve to yield insight into the complexity of accountability relations and the way the different components in the process of accountability can relate to each other. The application of agency theory to two cases in this study has also served to highlight its use and limitations. These insights will be discussed and elucidated below in the context of study findings from other settings and relevant conceptual tools and theory.

Despite their different socio-economic, historical and political contexts the qualitative data suggests that in both districts relationships between key governance actors are fraught with generally similar constraints on accountability for equitable and quality service provision as proposed in the triadic accountability model. This however does not exclude the existence of accountability relationships with a different nature. In the face of weak or absent formal accountability mechanisms it appeared that informal interpersonal and inter-organisational behaviours play an important role in establishing an accountability relationship, which confirms theoretical reflections in the field of (health) governance and accountability [23, 34–36]. These formal and informal accountability relationships form a complex institutional web in which agents sometimes also act as principals and vice versa. Particularly health providers, as street-level bureaucrats [37], find themselves to be in this role, being held accountable “from bottom-up, top-down as well as sideways” [38] as they face (sometimes conflicting) demands from state actors, citizens and development agencies.

In the relationship between health providers and state actors the findings suggest accountability for the delivery of the BBP is limited by insufficient resources to carry out this mandate, a rigid resource allocation rationale that is de-coupled from population needs or provider performance and monitoring activities that appear more aimed at finding faults in record-keeping and opportunities for resource-extraction through fines and (informal) co-payment revenue than at support for service delivery. This rent-seeking behaviour, which was reported in both districts irrespective of the co-payments associated with the BBP pilot, is in line with patterns in the wider bureaucracy as documented in a related study [14]. It is important to recognise that the negative, punitive character of this supervision style was found to be an important factor in health staff demotivation and attrition elsewhere and stands in contrast to the more supportive or coaching supervision approach by managers, which has been identified as a strong motivator for health workers in a broad variety of low and middle income settings [39–43]. The lack of decision space, limited resources and capacity to exercise effective accountability has also been found to be critical in other rural low resource settings [44, 45]. Its combination with a bureaucratically-engrained rent-seeking rationale, which turns monitoring and supervision into a power tool to incentivise the agents (health providers) to serve in the principal’s interest, particularly skews internal accountability away from provider performance, as has been documented extensively in India as well [46].

According to Hirschman [27] voice is one of the two important ways, together with *exit,* in which people respond to inadequate services. By extension Paul [47] considered them the two main factors that influence accountability. The findings of this study suggest that accountability between state authorities and citizens in the two districts is hampered by a disaffection among citizens with the severely limited opportunities for them to express their voice and the lack of effective formal enforceability mechanisms accessible to a wider public, i.e. a strong local legislative power that is chosen through free and fair elections. The lack of voice towards government actors resulting from a lack of belief in the possibility of answerability in Tajikistan corroborates findings from elsewhere in GBAO [48] and also echoes findings from other settings with recent experiences of authoritarian government [49]. This could also be a factor in explaining the lack of ‘rude accountability’ [50], found in this study. The instrumental use of threats through shaming or violence as a mechanism for frontline negotiations by citizens towards service providers, constituting this ‘rude accountability’, appears to be going hand in hand with a greater awareness of rights and rising expectations on social service provision in Bangladesh, where this was found [50]. Tajikistan markedly contrasts with this setting, as expectations of what the state is able or willing to deliver have massively reduced [51] and bureaucratic and democratic legitimacy have significantly eroded since the short-lived period of openness in the Soviet Union’s last years [52].

Based on the recognition that the ‘long route of accountability’ is often insufficient or ineffective in incentivising services to be more responsive (international) non-governmental organisations in comparable contexts have over the past decade initiated social accountability mechanism aimed at strengthening the ‘short route of accountability’ between citizens and health providers [6, 8]. In the two districts of this study however, the primary focus of the community-based organisations that have been formed, particularly in the GBAO district, is not on promoting active decision-making with state authorities. The degree to which they can enforce providers to be answerable appears to depend largely on ‘weak’ or ‘bridging’ social capital ties [53] that some community representatives manage to establish or nurture to voice their expectations and concerns [32], whilst information asymmetry between providers and citizens hampers the ability of the latter to do so. The in-kind ‘payments’ or support provided to local rural health facilities, in the GBAO district particularly, can be interpreted as a token in the creation of a social bond, or debt, with an obligation to reciprocate, as elaborated by Mauss and others [54], or as a limited form of co-production [55]. As Abimbola noted, this type of collective action by non-state (community) actors to keep primary healthcare services afloat can be found across LMICs, and can be seen as an informal example of collective governance [56].

The lack of strong or formal channels for citizens to voice their expectations or concerns around health services is particularly significant given the severely limited ‘exit options’ for people, particularly the poor [57]. Widespread poverty, geographical isolation, bad road infrastructure and a lack of private healthcare provision in addition to a limited network of public facilities contribute to this. The observation that both exit and voice options are severely limited can perhaps explain citizens’ efforts to contribute to the functioning of their local health centres. In other words, the lack of exit and voice options lock them in a type of continuing loyalty of ‘making do’ with the limited services that are available. This is a hypothetical inversion of Hirschman’s theory that high loyalty to a company, organisation or state works to limit people’s voice and exit options [27].

A number of limitations to the application of the triadic principal-agent model to the study of health systems of low-resource settings have surfaced in this study. First of all, the triadic model does not take account of the influence of the main external development agencies on citizens, providers and state authorities and the relationships between them in such settings. The findings from this study suggest the AKDN and project SINO have mainly dealt directly with health providers, establishing a principal-agent relationship parallel to that between state actors and health providers. This fits a pattern that donors in Tajikistan have mainly worked directly with beneficiaries instead of trying this through the government [58]. This pattern, although highly time-bound and likely to evolve, is unlike other fragile settings or areas of precarious statehood where external development actors and local health authorities engage in an unbalanced mutual dependency relationship [45] or networked governance of the health sector [59] in an imperfect attempt to foster local ownership and systems strengthening.

The cases explored in this study give insight into the internal divisions, power asymmetries and varying or sometimes competing interests, partly stemming from the inherited Soviet Semashko health system. It shows how complex practices of power and contestation over resources within the bureaucracy are influential in shaping policy implementation, mirroring the contestation over resources among local governance actors in South Africa [60]. The triadic model with its theoretically homogenous actors categories does not serve to understand these complex relations of power. Heterogeneity and competition within the actors categories was particularly evident in the ‘state’ and ‘providers’ groups. The competing interests of the Hospital Director, PHC manager, district health team and the district financial department revolved largely around access to and autonomy in decision-making over the allocation of scarce funds. The side-lining of the district health team in both districts, which was facilitated by the elusive formal mandate of this body and the double hat worn by the Hospital Director, as head of the most important health provider in the district, and key local government health official are the most striking examples of this. These examples confirm that the idea of holding a single agent category, such as ‘the state’, to account can be problematic in practice, summed up as ‘the problem of many hands’ that have contributed to any policy (outcome) [61].

Altogether these limitations throw up some fundamental question on the use of principal-agent theory for analysing health governance*.* Not only are the relations between actors more complex than suggested in the triadic model, the uncovering of rent-seeking rationales and co-production initiatives at community level suggest a need for other concepts and tools to explore practices of power, contestation and collaboration in local health governance. The application of principal-agent theory to governance has been criticised for ‘theoretically mischaracterising’ governance problems assuming the existence of ‘principled principals’, who are willing to hold agents to account whilst embodying the public interest, and the emphasis on individual incentive calculations [62]. This fails to recognise the implication of principals themselves in abuse of power for private gain, which is suggested in this study, and the expectation of others to be implicated in that. This influence of the (expected) behaviour of others on individual behaviour highlights the collective, rather than individual nature of rent-seeking and the importance of collective norms in perpetuating behaviour that is irrational from the viewpoint of the public good. Approaching governance as a collective action phenomenon, centred around the question what factors can help to overcome harmful equilibria of particularistic interests dominating in governance has therefore been seen as a more useful approach [63, 64]. Ultimately, however, the two approaches can also be considered complementary, as Marquette and Peiffer argue [65]. As this study shows, the application of principal-agent theory in the study of local health governance helps to unpack the incentives under which key stakeholders in the system relate to each other. It has provided insight into the challenges to the different components making up an effective accountability relationship, such as an unclear mandate, the lack of effective channels for voice or insufficient resources to carry out a mandate. Future research could help to further explore the phenomena found in this study, with attention to the function and the role of norms in rent-seeking behaviour, as well as the use of collective action theory to understand the role of (mis)trust in governance relations.

### Research limitations

This study has been subject to several limitations and its results are time-bound to the period of field research. Policy and organisational details have changed since data collection, and will continue to change as lessons are drawn, funding cycles change and new reforms are piloted or implemented. The limitations in data collection pertain to the relatively short period in which data collection took place, and the limited number of FGDs with citizens. Future studies could rely more on longer-term immersion and participant observation to better flesh out the complexities of agent’s motivations, strategies and practices of power. Respondent bias cannot be ruled out as AKDN, SDC’s project Sino and WHO facilitated entry to the study settings, although their representatives were excluded from interviews and FGDs with other stakeholders. Together with the closed political environment this may have biased respondents’ answers. Some limitations pertain to the topic of inquiry itself. Exploring power relations is sensitive in any setting, but particularly in an authoritarian environment with a legacy of large statist dominance of basic services, the economy and society [66] that has been penetrated by a more patrimonial type of governance by central elites [52, 67]. This requires provisions in the presentation of results to protect informants. Lastly, quantitative methods could have helped to gather more representative views on the daily practices of health workers and citizen’s voice.

## Conclusions

This qualitative study has presented an analysis of health governance at the district level in Tajikistan through an exploration of the way in which the main health system actors engage in a principal-agent relationship. In the application of the principal-agent model of health governance to district level Tajikistan, it has explored the previously understudied area of local health governance in post-Soviet Central Asia. This has highlighted the weak position of health providers and citizens vis-à-vis state actors and development agents and the prevalence of parallel lines of accountability towards health providers from development agents next to those assumed by the triadic accountability model. With consideration for the political and economic context the study also reveals the contestation over resources and resultant power play among local government actors and the existence of informal enforcement tools shaping de facto accountability relations. It thereby also served to demonstrate the limitations of this model in the study of health governance. This encourages further reflection on the complementarity of analytical concepts and tools from power and collective action theory and their application to the health system. More concretely, the study shows that particular attention needs to be paid to the importance of entrenched positions of power when introducing a new governance structure such as a district health team.

## Data Availability

Not applicable.

## Abbreviations

AKDN: Aga Khan Development Network
AKHS: Aga Khan Health Services
BBP: Basic Benefit Package
FGD: Focus group discussion
GBAO: Gorno Badakhshan Autonomous Oblast
KII: Key informant interview
MSDSP: Mountain Societies Development Support Programme
PHC: Primary healthcare
RRP: Districts of Republic Subordination (*Rayoni Respublikanskogo Podcinenja)*
VO: Village Organisation

## Footnotes

1 Between TJS 200 for a nurse and TJS 400 for a head doctor according to their own reports. At the time of research TJS10 = EUR 1.49, GBP 1.29, and US$ 2.12
